# Antiviral activity of myricetin glycosylated compounds isolated from *Marcetia taxifolia* against chikungunya virus

**DOI:** 10.17179/excli2023-6242

**Published:** 2023-07-27

**Authors:** Ana Luisa Muñoz, Andrés Felipe Cuéllar, Gabriela Arévalo, Brian David Santamaría, Anny K. Rodríguez, Cristian Buendia-Atencio, Andrés Reyes Chaparro, Aldo Yair Tenorio Barajas, Nidya Alexandra Segura, Felio Bello, Alírica I. Suárez, Héctor R. Rangel, Monica Losada-Barragán

**Affiliations:** 1 Faculty of Science, Universidad Antonio Nariño (UAN), Bogotá 110231, Colombia; 2 Escuela Nacional de Ciencias Biológicas (ENCB), Departamento de Morfología, del Instituto Politécnico Nacional (IPN), Mexico; 3 Facultad de Ciencias Físicomatemáticas, Benemérita Universidad Autónoma de Puebla C.U. Puebla, Puebla, Mexico; 4 Faculty of Science, Universidad Pedagógica y Tecnológica de Colombia, Tunja 150003, Colombia; 5 Faculty of Agricultural and Livestock Sciences, Program of Veterinary Medicine, Universidad de La Salle, Bogotá 110141, Colombia; 6 Natural Products Laboratory, Faculty of Pharmacy, Universidad Central de Venezuela, Caracas, Venezuela; 7 Molecular Virology Laboratory, Instituto Venezolano de Investigaciones Científicas, Caracas, Venezuela

**Keywords:** chikungunya, Marcetia taxifolia, cytotoxicity, antiviral activity, myricetin, molecular modeling

## Abstract

The chikungunya virus (CHIKV) has produced epidemic outbreaks of significant public health impact. The clinical symptoms of this disease are fever, polyarthralgia, and skin rash, generally self-limiting, although patients may develop a chronic disabling condition or suffer lethal complications. Unfortunately, there is no specific treatment or vaccine available. Thus, the search for effective therapies to control CHIKV infection is an urgent need. This study evaluated the antiviral activity of flavonoids isolated from *Marcetia taxifolia *by *in vitro *and *in silico *analysis. Cytotoxicity of compounds was determined by MTT assay and viral load was assessed in cell substrates supernatants by plaque-forming and RT-qPCR assays. Selected molecules were analyzed by molecular docking assays. Myricetin 3-rhamnoside (MR) and myricetin 3-(6-rhamnosylgalactoside) (MRG) were tested for antiviral assays and analyzed by the TCID50 method and RT-qPCR. MR exhibited dose-dependent antiviral activity, reducing viral titer at concentrations of 150-18.8 μg/mL by at least 1-log. Similarly, MRG showed a significant decrease in viral titer at concentrations of 37.5, 9.4, and 2.3 μg/mL. RT-qPCR analysis also displayed a substantial reduction of CHIKV RNA for both flavonoids. Furthermore, molecular docking of the selected flavonoids proposed the nsP3 macrodomain as a possible target of action. Our study reveals that MR and MRG could be considered promising anti-CHIKV therapeutic agents. Molecular modeling studies showed MR and MRG ligands with a high affinity for the N-terminal region of the nsP3 macrodomain, postulating them as a potential target of action for the CHIKV control.

## Introduction

Chikungunya virus (CHIKV) was discovered in East Africa in 1950 and was first characterized in Tanzania in 1952 (WHO, 2022[57]). Between 1990 and 2000, CHIKV re-emerged worldwide, affecting millions of people (Leparc-Goffart et al., 2014[34]). The first CHIKV outbreak in the Americas occurred in 2013, with more than 2.5 million people infected, accompanied by confirmed cases in Europe (Bonilauri et al., 2008[10]). The resurgence of CHIKV has been attributed to the spread of vectors to urban areas with poor hygienic conditions, climate change, and accessibility to global transportation systems (Gould et al., 2017[20]).

CHIKV (*Alphavirus* genus, *Togaviridae* family) is a positive-sense single-stranded RNA virus transmitted to humans by the bite of mosquitoes of the *Aedes aegypti *and *Aedes albopictus* species (Kantor, 2018[27]). However, vector control strategies to reduce viral spread have been ineffective; this is due in part to the selection, over different spraying campaigns, of organisms that exhibit resistance to commercially available insecticides. In addition, unsanitary conditions persist, generating abundant hotspots for vector proliferation (Suwanmanee and Luplertlop, 2017[53]).

Chikungunya fever presents a high degree of morbidity with a significant impact in developing countries. The infection is characterized by joint pain (arthralgia), myalgia, fever, nausea and headaches, which may resolve naturally. However, a high proportion of patients developed musculoskeletal disorders that persisted for months or years, probably due to a severe immune response (Restrepo et al., 2022[50]). Less frequently, this infection is associated with increased neurological damage and inflammatory activation of astrocytes in the gray matter of the brain (Kaur and Chu, 2013[28]). Nonetheless, there is currently no approved specific antiviral therapy or vaccine for CHIKV infection (DeFilippis, 2022[15]; Flipse and Smit, 2015[17]). Treatments are usually symptomatic and focus on alleviating symptoms. Unfortunately, side effects, such as gastrointestinal, hepatic, and renal toxicity, Reyes syndrome and bleeding, have been reported (Brito et al., 2016[11]; Kaur and Chu, 2013[28]). Therefore, it is essential to support new strategies for developing specific antiviral therapies and vaccines against this etiological agent (Deeba et al., 2016[14]; Hucke et al., 2021[22]). 

Natural products extracted from plants are considered one of the most critical sources of compounds with biological effects, including antiviral activities (Ninfali et al., 2020[42]). *Marcetia* is a neotropical genus belonging to the Melastomataceae family, distributed in tropical and mountainous areas of South America, Southeast Asia, and Southern China (Rocha et al., 2016[51]), with 44 described species (Liogier, 2000[36]). *Marcetia* *taxifolia* is a shrub distributed in Colombia, Venezuela, Guyana, and Brazil (Leite et al., 2012[33]). Extracts and compounds isolated from this plant have displayed antiviral, antioxidant, antibacterial, and anti-inflammatory activities (Leite et al., 2012[33]). The compounds responsible for these effects are flavonoids such as myricetin, quercetin and pinocembrin, present in most plant tissues, and show low toxicity in eukaryotic cells (Leite et al., 2012[33]).

Myricetin and quercetin have been shown to interfere with HIV-1 proliferation by inhibiting reverse transcriptase (RT) enzymatic activity (Pasetto et al., 2014[47]). This enzyme plays an essential role in the HIV-1 life cycle of and is one of the main targets of several anti-HIV-1 drugs in clinical use (Jonckheere et al., 2000[26]; Mansouri et al., 2023[38]; Pasetto et al., 2014[47]). Likewise, compounds derived from quercetin modified by a glycoside substituent showed antiviral activity by RT inhibition (Ortega et al., 2017[45]). Furthermore, *in silico* studies have successfully demonstrated how the interaction of active compounds from *M. taxifolia* against NS3-helicase and NS5-RNA polymerase of ZIKV and DENV can be used as potential inhibitors of infection and, therefore, for the development of specific antiviral agents (Buendia-Atencio et al., 2021[12]).

Moreover, the CHIKV nsP3 has been implicated in virus-protein-host protein interaction in the early stages of the viral replication cycle. Furthermore, it has been identified as crucial for viral RNA replication, translation, and virulence (Zhang et al., 2021[60]). Consequently, it is a potential target for drug development against CHIKV infection. 

This study evaluated the efficacy of myricetin glycosylated compounds extracted from the *M. taxifolia* plant as potential antiviral agents against CHIKV *in vitro*. In addition, the interaction of MR and MRG compounds from* M. taxifolia* with the N-terminal region of the nsP3 of CHIKV was determined by docking and molecular dynamics. 

## Materials and Methods

### Cell line culture and viral propagation

Vero (African green monkey kidney fibroblasts, ATCC-CL 81) and BHK-21 (Baby Hamster Kidney-21, ATCC-CCL 10) cell lines were used for viral infection. They were maintained in Dulbecco's modified minimal essential medium (DMEM - Lonza®, Catalogue No. 12-604Q), supplemented with 10 % fetal bovine serum (FBS) (Biowest®, Catalogue No. S18b-500), and 1 % of penicillin-streptomycin antibiotic solution (Lonza®, Catalog No.17-602F) and incubated at a temperature of 37 °C with a 5 % CO_2_ atmosphere. The CHIKV strain was isolated from Colombian clinical samples and characterized by RT-qPCR assays. Viral stocks were prepared by infecting Vero cells to a third passage; briefly, cell supernatants were harvested when 70 % of the cells showed a cytopathic effect, aliquoted, stored at - 80 °C, and used throughout the study. After the virus was propagated, the infectivity titer was determined by inoculation with increasing dilutions of the virus (10^−1^ up to 10^−10^) into Vero cells, grown to confluent monolayer in 96 well plates, in eight replicates. After three days of incubation, the highest dilution showing 50 % infection was used to calculate the TCID50/mL by the Reed-Muench formula (Reed and Muench, 1938[49]).

### Plant material and preparation of vegetal flavonoids

The plant *Marcetia taxifolia* (A.St.-Hil.) was collected in the Amazonas State of Venezuela and archived (MYF 28418) in the "Herbario Víctor Manuel Ovalles" (Victor Manuel Ovalles Herbarium) of the Pharmacy school of the Universidad Central de Venezuela. The compounds of *M. taxifolia* were extracted and purified as previously described (Baptista et al., 2016[7]). Briefly, the plant parts were dried, macerated, and separated by organic extraction. The isolated flavonoids were fractionated by chromatographic techniques and characterized by spectroscopic and spectrometric methods. The stock solution of myricetin 3-(6-rhamnosilgalactoside) (MRG), 5,3'-dihydroxy-3,6,7,8,4'-pentamethoxyflavona (PMF), 5-hydroxy-3,6,7,8,3',4'-hexamethoxyflavona (HMF), and myricetin rhamnoside (MR) were prepared by dissolving each compound in DMSO to a final concentration of 10 mg/mL. Subsequently, the stock solutions were stored at -20 °C until use. Further dilutions were made in DMEM at the time of use.

### Viral susceptibility assay

To establish the cell line most susceptible to CHIKV growth, 50.000 BHK-21 cells/well and 35.000 Vero cells/well were plated into 96-well plates 16 to 24 h prior to infection. The culture medium was then removed, and cell monolayers were washed twice with 1X PBS (Lonza®, Catalogue No. 17-516Q). Cells were infected with CHIKV at a multiplicity of infection (MOI) of 0.0001 for one hour for viral adsorption. The cell monolayers were then washed thrice with 1X PBS to remove the non-adsorbed virus. Finally, 200 μL of DMEM 2 % FBS was added, and incubated for up to 106 hours at 37 °C in 5 % CO_2_. At 10, 22, 34, 46, 63, 70, and 106 hours, supernatants and infected cells were collected and stored at -70 °C for quantification by plaque assay and RT-qPCR. The plaque assay was performed by seeding 70.000 BHK-21 cells/mL; 16 hours after, each supernatant was serially diluted from 10^-1^ to 10^-8^ in DMEM supplemented with 2 % SFB (2 % DMEM). Three replicates were performed for each dilution. After one hour of viral adsorption, 1.5 % of carboxymethyl cellulose (CMC) in 2 % DMEM was added to each well. The plates were then incubated in 5 % CO_2_ at 37 °C for five days, after which the medium was removed, and the cells were fixed with Crystal Violet staining solution (3.5 % formaldehyde, 0.2 % w/v cristal violet) for one hour. The solution was then removed, the plates were washed with water, dried at room temperature, and finally the lysis plaques formed were counted to calculate the viral titer by adding 200 μL of assay medium. The cultures were incubated for five days and subsequently fixed with formaldehyde, stained with crystal violet and the plaques were counted to determine the viral titer expressed in plaque-forming units (PFU/mL) (Alvarez et al., 2005[5]).

### Cytotoxicity assay of M. taxifolia flavonoids

The cytotoxicity of *M. taxifolia* flavonoids was assessed on BHK-21 cells by performing an MTT assay. Briefly, cells were seeded in 96-well plates at a cell density of 30.000 cells/well and incubated for 24 h at 37 ° C. Cells were incubated with serial dilutions ranging from 250 to 0,24 μg/mL of each of the four compounds for 24, 48, and 72 h at 37 °C with three replicates set up in each group. Cells were then incubated with 10 μL of MTT reagent (Roche®, Catalogue No. 11465007001) for an additional 4 h at 37 °C. The formazan crystals were solubilized in DMSO and measured using the Multiskan^TM^ FC microplate reader (Thermo Scientific) at 570 nm. Percentage cytotoxicity or viability was calculated compared to untreated cells. In addition, the concentration that inhibited 50 % of cell growth (CC50), which reduces the metabolic activity of cells by 50 %, was calculated by logarithmic interpolation, following a previously described procedure (Żesławska et al., 2018[59]).

### Antiviral assay of M. taxifolia flavonoids

To determine the antiviral activity of MR and MRG on CHIKV, BHK-21 cells were used. For this purpose, 30,000 cells were seeded in each well of a 96-well plate and incubated for at least 16 hours at 37 °C 5 % CO_2_. Subsequently, two-fold serial dilutions obtained from the CC50 of MR and MRG (150 to 1.17 μg/mL for MR and 75 to 0.58 μg/mL for MRG) were added in triplicate. Then, 100 μl of a CHIKV suspension was added as described above and incubated for 72 h. The cell control was untreated and non-infected cells, and the virus control wells were untreated but infected cells (with the same virus concentration). No pharmacological control was used as there are no specific antiviral drugs approved to treat this infection. After treatment, the infection was allowed to proceed for 72 hours and cells were observed for the presence of the cytopathic effect (CPE). At the end of incubation, culture supernatants were collected, divided, and analyzed by the Reed and Muench method (Reed and Muench, 1938[49]) and RT-qPCR, as previously described.

### One-step RT-qPCR 

CHIKV RNA copy number was determined using a quantitative RT-qPCR approach. Viral RNA was isolated from previously infected Vero or BHK-21 cells using a commercial viral RNA extraction kit according to the manufacturer's instructions (BMG-VR6568-02 Biomiga Inc). For RT-qPCR, a one-step PCR was performed at a final volume of 5 μL containing: 1 ng/μL of RNA per reaction, 2.5 μL of 2X qMax Green One-Step Mix, 0.25 µL of 20X RTase Blend, and 0.4 μM of each primer. Primers were specific to the CHIKV unique domain of nsP1 (CHIKV-F 5'-ACGTGGATATAGACGCTGACAG-3' and CHIKV-R 5'-GCATGGTCATTTGATGTGACC-3'). Amplification was performed using the QuantStudio 3 thermal cycler (Applied Biosystems) under the following thermal cycling conditions: 1 cycle at 50 °C for 10 min for reverse transcription, one cycle at 95 ° C for 2 min for initial denaturation and polymerase activation, and 40 cycles of 95 °C for 5 sec, annealing temperature at 62 °C for 30 sec, and elongation at 72 °C for 30 sec. A melting curve analysis was then performed at 65 °C to 95 °C to verify the assay's specificity. During the runs, two negative controls were included. In the first, the RNA sample was replaced with RNase-free water, and in the second, RNA from uninfected cells was used. The absolute amounts of viral RNA were calculated with a standard curve generated with a 10-fold serially diluted viral RNA extracted from CHIKV inoculum of known titer at 3.16 x10^8^ TCDI50/mL. All samples were analyzed in duplicate.

### Molecular docking and dynamics 

Molecular docking studies were carried out with the "X" domain, known as the macrodomain, present in viruses of the Coronavidiridae and Togaviridae families, which is activated as adenosine diphosphoribose phosphatase in Chikungunya viruses (Malet et al., 2009[37]). The nsP3 macrodomain (PDB 3GPG) was selected for molecular docking, and MR and MRG compounds were isolated from *M. taxifolia*.

For the nsP3 macrodomain, a grid box of dimensions 20x, 22y and 20z (Å) with a spacing of 1 Å was defined. The 3GPG crystallographic structure had a resolution of 1.8 Å and adequate metric validation. Calculations with the MR and MRG ligand structures defined these lattice box dimensions as the most accurate docking parameters. The 3D structures of MR and MRG were obtained using the PubChem database (Kim et al., 2016[30]), and their charges were added using the Biovia Discovery Studio (3DS, 2020[1]). Molecular docking was assessed using AutoDock Vina (ADV) software (Morris et al., 2009[40]). A maximum energy difference of 5 kcal/mol between the best and worst poses and a completeness of 8, which is the number of evaluations that take place in the local optimization of a conformer, were used.

Molecular dynamics assays were performed to assess the stability of the ligand-receptor complexes resulting from the molecular docking assays. The CHARMM-GUI platform was used to prepare the different inputs, and the Gromacs 2021.1 software was used for molecular dynamics (Abraham et al., 2015[3]; Jo et al., 2008[25]; Lindahl et al., 2021[35]). Each protein was preprocessed using the PDB reader tool (Jo et al., 2014[23]). On the other hand, the resulting docking ligands with the highest affinity energy were selected and shifted to mol2 format using OpenBabel (O'Boyle et al., 2011[43]). Next, the ligand .mol2 files were loaded into the Ligand Reader & Modeler tool to generate the parameters and topology files (Kim et al., 2017[29]). Finally, the ligand-receptor complexes were integrated into a single .pdb file that was used in the “Solution Builder” tool to create the system used as input for Gromacs (Lee et al., 2016[32]). The water box was cubic, fitted to the protein size and with 10 Å edge spacing. Each system was neutralized using KCl ions placed by the Monte-Carlo method at a concentration of 0.15 M. Each system was subjected to 5000 steps of energy minimization of steeper decays to eliminate the steric overlap. Then, all systems were subjected to an NVT (constant number of particles, volume and temperature) equilibrium phase for 125000 steps, using the V-rescale temperature-coupling method, with a constant coupling of 1 ps at 303.15 K (Bussi et al., 2007[13]). Subsequently, molecular dynamics was carried out for 100 ns using the CHARMM36m force field (Vanommeslaeghe et al., 2010[55]). Gromacs utilities were used to evaluate the root-mean-square deviation (RMSD) of complexes, and that of each protein and ligand, the root-mean-square fluctuation (RMSF) and hydrogen bonds. The data were plotted using the GRACE program. 

### Statistical analysis

Data were analyzed with Student´s t-test and One-way ANOVA test using GraphPad Prism Software version 8.4.0. A p-value < 0.05 was considered statistically significant. All experiments were performed at least three times with three replicates. For each experiment, the mean and standard deviation of the mean are shown. The RT-qPCR data from antiviral assays were transformed using a base 10 logarithm. Then, using the dose-response algorithm, the Ct obtained was normalized to determine the effective concentration 50 (EC50).

## Results

### Susceptibility of BHK-21 and Vero cells to CHIKV

**Figure 1:**
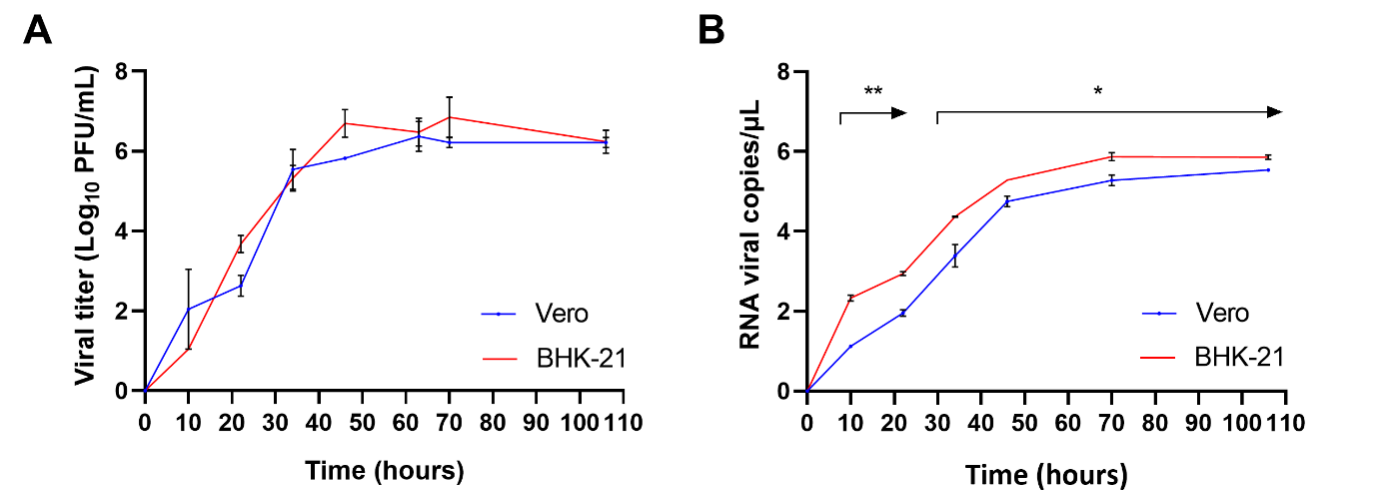
Susceptibility of Vero and BHK-21 cell lines to CHIKV infection. Vero and BHK-21 cell lines were inoculated with CHIKV at MOI of 10^-4^. Culture supernatants and infected cells were collected at 10, 22, 34, 46, 63, 70, and 106 hours. (A) Plaques were counted in plates previously seeded with cells incubated with the collected medium for five days. (B) RNA from infected cells was extracted, and the nsP1 gene was amplified by RT-qPCR. Data are presented as the mean and standard deviation of the mean (SEM). Statistical differences were analyzed using a student t-test *p<0.05, **p<0.005.

To evaluate the viral susceptibility of BHK-21 and Vero cells to the Colombian CHIKV strain, plaque and RT-qPCR assays were performed. CHIKV replicated in the two cell lines with similar log growth rates, indicating that the viral yield increased during the incubation (Figures 1A and B(Fig. 1)). Growth kinetics analyzed by plaque-forming unit assay showed no statistical difference between viral yields in both cell lines, maintaining ∼6 log viral titer up to 106 hours post-infection (Figure 1A(Fig. 1)). Likewise, replication kinetics assessed by RT-qPCR showed an increase in viral yield in both cell lines over time (Figure 1B(Fig. 1)). Notably, viral RNA copies detected in BHK-21 were significantly higher than those in Vero cells at all time points analyzed (Figure 1B(Fig. 1)), indicating that the BHK-21 cell line is more sensitive to CHIKV infection than Vero cells and support a higher level of CHIKV RNA replication.

### Cytotoxicity of M. taxifolia flavonoids on BHK-21 cells

Before determining the antiviral activity of *M. taxifolia* flavonoids, BHK-21 cells were used to evaluate their cytotoxic effects, taking into account the higher susceptibility of BHK-21 cells compared to the Vero cell line. HMF and PMF exhibited cytotoxic activity toward BHK-21 cells even with exposure to low concentrations of each compound (Table 1(Tab. 1)). HMF displayed the highest cytotoxicity with the lowest CC50 value at 48 (4.98 μg/mL) and 72 hours (5.4 μg/mL) among all flavonoids tested. PMF also showed high cytotoxic action for BHK-21 cells with a CC50 value of 34.94 and 43.61 μg/mL at 48 and 72 hours, respectively. In contrast, no significant cytotoxic effect was observed after exposure of BHK-21 cells to MR and MRG concentrations up to 250 μg/mL. Therefore, MR and MRG were selected to study their antiviral activity against CHIKV.

### Antiviral activity of M. taxifolia flavonoids against CHIKV

**Figure 2:**
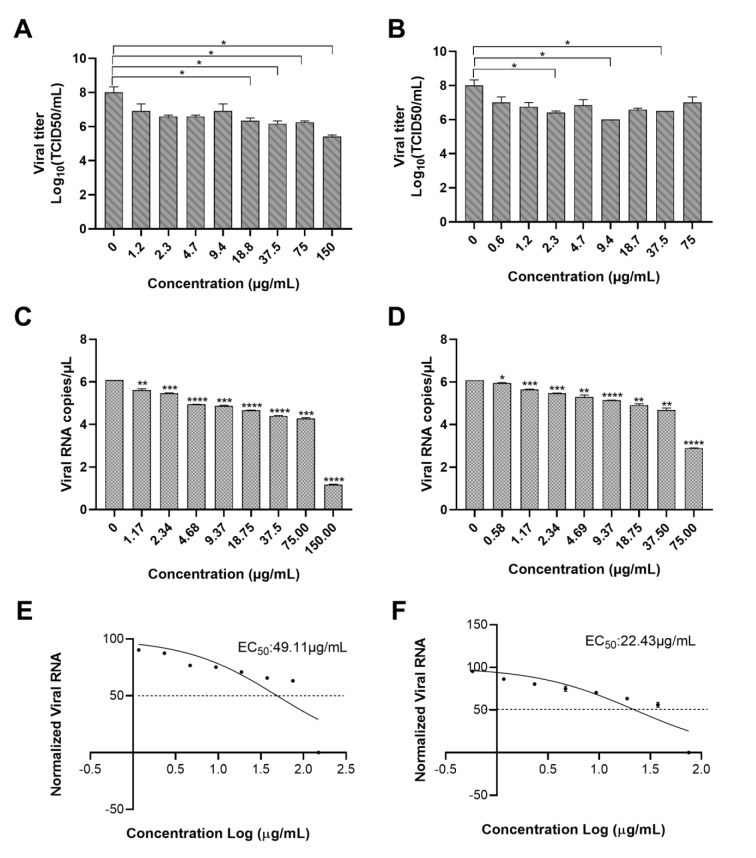
Antiviral activity of MR and MRG against CHIKV. BHK-21 cells infected with CHIKV were exposed to serial dilutions starting from 150 to 1.17 µg/mL for MR (A) and 75 to 0.58 µg/mL for MRG (B) for 72 hours. Viral titer was obtained after five days of cell incubation by the Reed and Muench method. Viral titer data are presented as log10 titer mean ± standard deviation of the mean (SEM). Statistical differences were analyzed using a student t-test (*p<0.05). nsP1 viral RNA copies from infected cells incubated with MR (C) or MRG (D) were amplified by RT-qPCR. Data were transformed using a base 10 logarithm and analyzed using a student t-test (*p<0.02, **p<0.05, ***p<0.001, ****p<0.0001). Using the dose-response algorithm, the Ct obtained was normalized to determine the effective concentration 50 (EC50) for MR (E) or MRG (F).

Viral titer and nsP1 RNA copy numbers were determined to evaluate the antiviral activity of MR and MRG against CHIKV after 72 hours of treatment. BHK-21 cells infected with the Colombian strain of CHIKV were exposed to MR or MRG in a concentration range of 150 to 1.17 μg/mL for MR and 75 to 0.58 μg/mL for MRG, obtained from the CC50. Remarkably, both flavonoids exerted an important antiviral activity toward CHIKV. MR exhibited a dose-dependent antiviral activity, significantly reducing viral titers with doses from 150 to 18.8 μg/mL (Figure 2A(Fig. 2)). Similarly, MRG showed a significant decrease in the viral titer at a concentration of 37.5, 9.4 and 2.3 μg/mL (Figure 2B(Fig. 2)). Exposure to each compound for 72 h post-infection resulted in a reduction of viral titer from 8.00 to 5.42 log10 TCID50/mL mean at 150 μg/mL for MR and a reduction from 8.00 to 6.50 log10 TCID50/mL mean at 37.5 μg/mL.

Consistent with these findings, RT-qPCR analysis also showed a significant decrease in CHIKV RNA log copy number for MR and MRG treatments at all concentrations tested compared to untreated cells in a dose-dependent manner (Figure 2C and D(Fig. 2)). MR dramatically reduced viral load (80.78 ± 0.46 %) at 150 μg/mL, whereas MRG decreased by only 52.46 ± 0.12 % at the highest concentration tested. However, when we determined the effective concentration (EC50) of each flavonoid against CHIKV by RT-qPCR (Figure 2E and F(Fig. 2)), MR showed a 50 % higher effective concentration (EC50) (49.11 ± 0.12 µg/mL) than MRG (22.43 ± 0.69 µg/mL), suggesting that MRG is a most effective antiviral compound. 

### Molecular modeling of MR and MRG with nsP3

**Figure 3:**
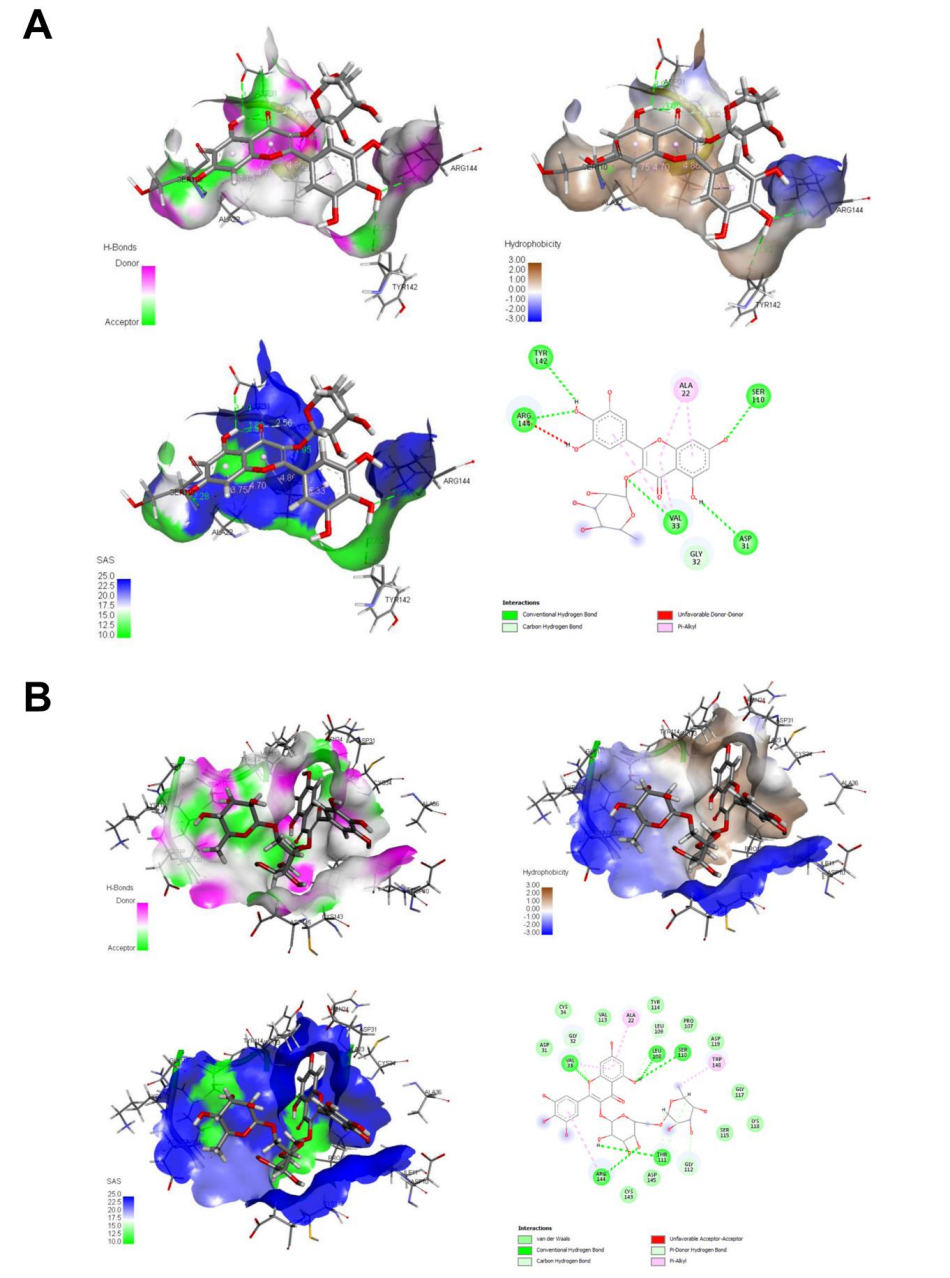
MR and MRG docked with nsP3 protein, 2D and 3D representation. A) MR-nsP3 complex, affinity energy of -9.1 kcal/mol. B) MRG-nsP3 complex, affinity energy of -8.5 kcal/mol. Molecular docking assays were carried out with AutoDock, 2D figures with PoseView of ProteinsPlus, and 3D figures with UCSF Chimera. Left superior figure presents H-bonds, right superior figure presents hydrophobicity, left inferior figure presents solvent accessibility and right inferior figure presents 2D interaction diagram.

**Figure 4:**
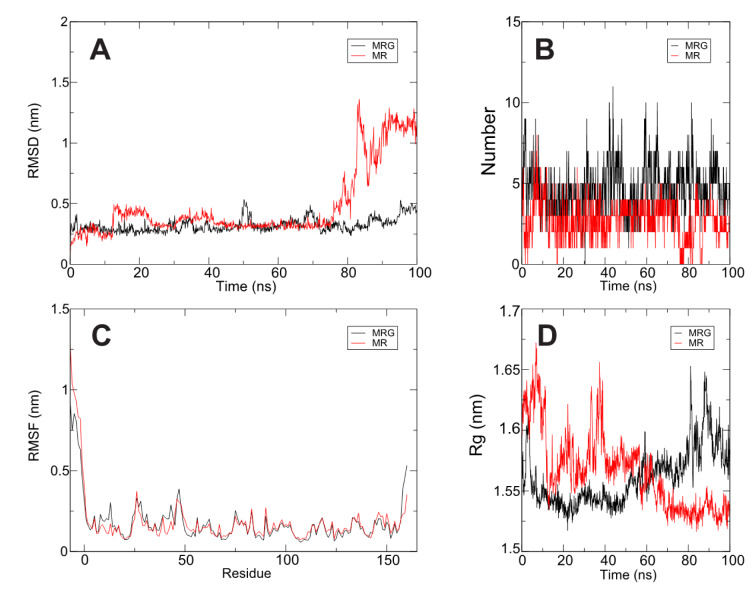
Molecular dynamics of MR and MRG-nsP3 complexes. A) RMSD Root Mean Square Deviation. B) H-bonds between ligand and protein. C) RMSF Root Mean Square Fluctuation of nsP3 protein. D) Radius of gyration. For additional information see Supplementary Figure 1 and 2.

Docking assays showed high affinity of MR and MRG with the nsP3 macrodomain, with an affinity energy of -9.1 and -8.5 kcal/mol, respectively. The MR ligand formed 6 hydrogen bonds with the residues: Ser110, Asp31, Val33, Arg144, and Tyr142, one Carbon-Hydrogen bond with Gly42, additionally 4 hydrophobic interactions with the Ala22 and Val33 residues (Figure 3(Fig. 3) and Table 2(Tab. 2)). Furthermore, the MRG presents 5 hydrogen bonds with residues Val33, Arg144, Thr111, Ser110, and Leu108, 6 Carbon-hydrogen bonds with Gly32, Leu109, Gly112, and Thr111, one Pi-donor-hydrogen bond with Val33 and 6 hydrophobic interactions with Val33, Ala22, Arg144 and Trp148 (Figure 4(Fig. 4) and Table 2(Tab. 2)). The stability of the complexes was confirmed by means of 100 ns of molecular dynamics, observing that the position of the ligand in the predicted binding site is maintained (Figure 4A(Fig. 4)). A twist in the MR ligand is observed at 80 ns, but it is a rearrangement and remains in the binding site; the MRG-nsP3 complex remains more stable throughout the simulation.

The stability of the MR and MRG is directly related to the ability to create hydrogen bonds, as can be seen in Figure 4B(Fig. 4), while the MR presents a lower amount of H-bond interactions. Figure 3(Fig. 3) shows the H-bonds formed by the ligand-receptor complex, and it is observed that the availability of -OH groups act both as donors and acceptors of H-bonds, while the protein presents interaction zones marked with red and blue colors for negative and positive polar interaction surface respectively. Finally, it is observed that the protein does not present relevant structural modifications as a result of molecular dynamics. The RMSF shows us the fluctuation of the position of the amino acids, and the whole protein shows a movement of less than 0.5 nm, except for the terminal groups that usually present a wide movement as they are disordered regions. This is also corroborated by the radius of gyration which has a fluctuation of less than 0.2 nm (1.5 to 1.7 nm), showing that the protein has not presented changes in size and conformation.

See also the Supplementary data and the Supplementary information. 

## Discussion

Chikungunya fever is a vector-borne disease that impacts millions of people worldwide. Despite advances in viral disease treatments, there are currently no antiviral therapies available to treat or prevent CHIKV infection. Flavonoids and related compounds have been isolated from different natural sources, revealing multiple biological functions, including antiviral properties (Badshah et al., 2021[6]; Kumar and Pandey, 2013[31]). 

Flavonoids can have modifications such as glycosylation in which different sugars can be found, such as glucose, rhamnose galactose, arabinose, and rutinose (Xiao, 2017[58]). This glycosylated part confers critical characteristics from a drug design point of view, such as solubility (Godinho et al., 2021[19]), better absorption, cellular recognition (Behl et al., 2021[8]), and decreased toxicity (Plaza et al., 2014[48]). In addition, it has been described that these flavonoids can be a substrate for sodium-dependent glycoside transporters (SGLT1) in the enterocyte membrane (Tapiero et al., 2002[54]), contributing to their cellular internalization. Based on these precedents, initially, we tested the cytotoxicity of four flavonoids isolated from the aerial parts of *M. taxifolia*. MR and MRG exhibited the lowest cytotoxic effects compared to the methoxyflavones HMF and PMF. The low toxicity of MR and MRG could be related to the glycosyl moieties present in their structure. Moreover, previous reports had associated the degree of glycosylation with inhibition of viral replication (Ortega et al., 2017[45]). Therefore, we selected these two myricetin glycosylated compounds to analyze their antiviral properties against CHIKV.

MR and MRG antiviral effects against different types of viruses are described. In particular, interesting properties against hepatitis B virus (HBV) (Ortega et al., 2019[44]; Parvez et al., 2020[46]), human immunodeficiency virus 1 (HIV-1) (Ortega et al., 2017[45]), influenza A virus (IAV) (Motlhatlego et al., 2021[41]), and African swine fever virus (ASFV) (Jo et al., 2020[24]) are described. However, they are ineffective against poliovirus (PV-1) and herpes simplex virus **(**HSV-1) (Ortega et al., 2019[44], 2017[45]). Notably, we also observed potent antiviral activity of MR and MRG compounds toward CHIKV without cytotoxic effects on the BHK-21 cell line. Based on viral yield in terms of viral RNA or viral titer, it could be observed that 18.8 μg/mL of MR and 2.3 μg/mL of MRG inhibited CHIKV replication efficiently with an EC50 <50 µg/mL, indicating that both flavonoids display a potent antiviral activity.

Some authors described that MR and MRG could act as non-nucleoside reverse transcriptase inhibitors (NNRTI), blocking the reverse transcriptase activity in HIV-1 (Ortega et al., 2017[45]). Similarly, the anti-HBV activity of MR has been mainly associated with binding to HBV polymerase/reverse transcriptase (Pol/RT) (Parvez et al., 2020[46]). However, the mechanism of action of both compounds in CHIKV must differ from those viruses, considering that CHIKV replication does not involve reverse transcriptase. An alternative mechanism for the antiviral effects of MR was proposed in a combined co-penetration treatment for IAV, in which MR caused an inhibitory impact on the viral attachment and viral entry stages of the virus (Motlhatlego et al., 2021[41]). Therefore, it may be possible that the anti-CHIKV properties of MR and MRG may also act through the initial stages of infection. In addition, the antiviral activity of MR and MRG could be related to the glycosyl moieties in the molecule, as we found that the MRG molecule with the most carbohydrate residues shows a more effective antiviral activity. However, more assays are needed to verify this hypothesis.

The use of myricetin and derived related compounds in the treatment of CHIKV provides additional benefits due to their multiple biological functions. These include anti-inflammatory effects (Hou et al., 2018[21]), enhanced immunomodulatory functions (Ghassemi-Rad et al., 2018[18]), reduction of oxidative stress (Bertin et al., 2016[9]; El-Haleem et al., 2016[16]), and amelioration of nervous system diseases (Ahmed et al., 2019[4]; Meyer et al., 2017[39]; Wang et al., 2019[56]), among others (Song et al., 2021[52]). Remarkably, chikungunya fever results in a number of clinical manifestations with an underlying proinflammatory profile in both the acute and chronic stages of the disease. Symptomatology such as fever, joint and muscle pain, skin rashes, arthralgia or chronic arthritis, musculoskeletal lesions, fatigue and neurocognitive impairment could be alleviated with these compounds. Though, further functional studies should be conducted to explore these additional properties of myricetin-related compounds in chikungunya fever disease.

The nsP3 macrodomain is crucial for viral RNA replication and translation. It is also involved in virus-host protein-protein interaction in the early stages of the viral replication cycle (Abdullah et al., 2021[2]). Sequence analysis of the structures of the macrodomains of Chikungunya Virus, Venezuelan equine encephalitis, SARS-CoV (2003 pandemic), MERS-Cov (2012 pandemic) and SARS-CoV-2 (2020 pandemic) was performed, looking for added value to the possible inhibitory effect of natural compounds on this common target. The alignments of the Venezuelan equine encephalitis virus macrodomain structure (PDB 3GQE) and the Chikungunya virus macrodomain region (PDB 3GPG) both of 168 residues were analyzed yielded a poor percentage of similarity, 11.90 %; identity, 5.9 %; and homology, 17 %. In the case of the alignments between the macrodomains of SARS-CoV nsp3 (PDB 2JZD); MERS-CoV (PDB 5HOL); SARS-CoV-2 macrodomain (NSP3) (PDB 6WOJ) and the Chikungunya macrodomain (3GPG) the % similarity, homology and identity was less than 30 %. For this reason, we ruled out the possibility of extrapolating the computational predictions to a possible inhibitory effect of natural compounds on this common target. Starting from this point, docking studies were performed for the ligands with lower cytotoxicity and higher percentage of *in vitro* inhibition of *M. taxifolia*, such as MR and MRG and the macrodomain region of nsP3 (PDB id 3GPG), the first 160 residues of the N-terminal region, obtaining MR results with interaction energy -9.1 and MRG with interaction energy -8.5, molecular dynamics tests were performed to evaluate the stability of the ligand-receptor complexes that resulted from the molecular docking tests, in their results it can be observed that the size varies between 1.68 and 1.52 nm, the interaction with the MR increases the radius of gyration. Still, it stabilizes around 40 ns and decreases until the end of the interaction. On the other hand, the interaction with MRG is increasing until the end of the simulation, it is not a significant increase, but this gives more evidence that the interaction with MR will be more stable. Therefore, it is possible to consider the nsP3 macrodomain as a potential target of action of these compounds.

Our current *in vitro* findings identify MR and MRG as promising antiviral flavonoids for treating CHIKV infection when added up to 72 h post-infection. Cytotoxicity analysis and antiviral assays demonstrate robust antiviral activity, with MRG acting more effectively than MR. Apart from the ability of MR and MRG to inhibit CHKV infection, their reported anti-inflammatory activities may also be of importance, as this may alleviate symptoms associated with CHIKV fever. In this work, molecular modeling studies showed the MR and MRG ligands with high affinity for nsP3, -9.1 and -8.5 kcal/mol, respectively, and by molecular dynamics at a time interval of 100 ns, the stability of the complex was confirmed. Thus, the N-terminal region of the nsP3 macrodomain is postulated as a potential target of action of CHIKV. However, further *in vivo* studies exploring its potential as a flavonoid antiviral and other beneficial side effects are essential.

## Declaration

### Acknowledgments

This work was supported by Minciencias under research grant No. 124380864546 - contract CT. FP 80740- 152-2019; Universidad Antonio Nariño (UAN) under grant No. 2022206 and its Young Researcher Program. All authors acknowledge to the editorial fund and the Vicerrectoría de investigación (VCTI) from the UAN. We are grateful to Professor Jaime Castellanos from Universidad El Bosque for providing the virus strain.

### Conflict of interest

The authors declare no conflict of interest.

## Supplementary Material

Supplementary data

Supplementary information

## Figures and Tables

**Table 1:**
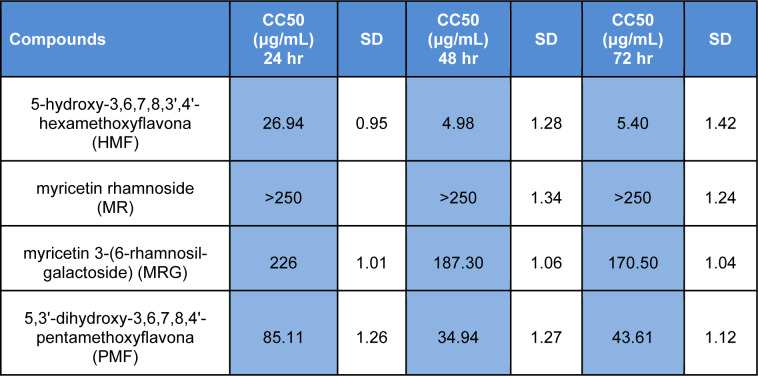
Cytotoxic activity of *Marcetia taxifolia *flavonoids on BHK-21 cell line

**Table 2:**
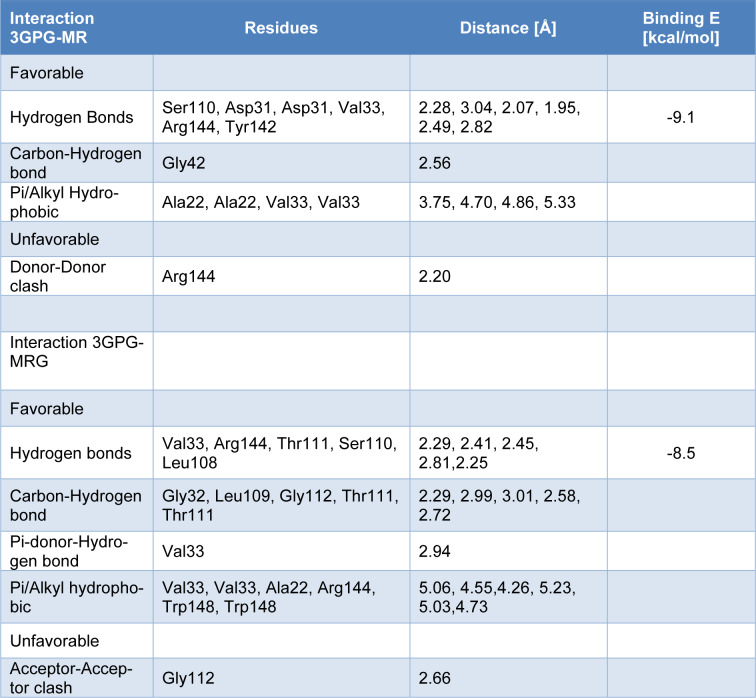
Favorable and unfavorable 3GPG-MR and 3GPG-MRG, protein-ligand interactions, distance in angstroms, total binding E kcal/mol from docking
