# Supplementary material for: Antiviral activity of myricetin glycosylated compounds isolated from Marcetia taxifolia against chikungunya virus
Source: EXCLI J. 2023 Jul 27;22:716–31. doi: 10.17179/excli2023-6242 (PMC10471840; doi:10.17179/excli2023-6242)
Supplement: Supplementary data [file EXCLI-22-716-s-001.pdf]

EXCLI Journal 2023;22:716-731 – ISSN 1611-2156
Received: June 08, 2023, accepted: July 20, 2023, published: July 27, 2023

Supplementary data to:

Original article:

ANTIVIRAL ACTIVITY OF MYRICETIN GLYCOSYLATED COMPOUNDS ISOLATED FROM *MARCETIA TAXIFOLIA* AGAINST CHIKUNGUNYA VIRUS

Ana Luisa Muñoz[1], Andrés Felipe Cuéllar[1], Gabriela Arévalo[1], Brian David Santamaría[1], Anny K. Rodríguez[1], Cristian Buendia-Atencio[1], Andrés Reyes Chaparro[2], Aldo Yair Tenorio Barajas[3], Nidya Alexandra Segura[4], Felio Bello[5], Alírica I. Suárez[6], Héctor R. Rangel[7], Monica Losada-Barragán[1*]

[1] Faculty of Science, Universidad Antonio Nariño (UAN), Bogotá 110231, Colombia
[2] Escuela Nacional de Ciencias Biológicas (ENCB), Departamento de Morfología, del Instituto Politécnico Nacional (IPN), Mexico
[3] Facultad de Ciencias Físicomatemáticas, Benemérita Universidad Autónoma de Puebla C.U. Puebla, Puebla, Mexico
[4] Faculty of Science, Universidad Pedagógica y Tecnológica de Colombia, Tunja 150003, Colombia
[5] Faculty of Agricultural and Livestock Sciences, Program of Veterinary Medicine, Universidad de La Salle, Bogotá 110141, Colombia
[6] Natural Products Laboratory, Faculty of Pharmacy, Universidad Central de Venezuela, Caracas, Venezuela
[7] Molecular Virology Laboratory, Instituto Venezolano de Investigaciones Científicas, Caracas, Venezuela

* **Corresponding author:** Dra. Monica Losada-Barragán, Universidad Antonio Nariño-Sede Circunvalar. Cra. 3 este # 47A - 15, Bogotá, Colombia.
E-mail: monica.losada@uan.edu.co

https://dx.doi.org/10.17179/excli2023-6242

This is an Open Access article distributed under the terms of the Creative Commons Attribution License (http://creativecommons.org/licenses/by/4.0/).

EXCLI Journal 2023;22:716-731 – ISSN 1611-2156
Received: June 08, 2023, accepted: July 20, 2023, published: July 27, 2023

Raw data concerning **Figure 1:** Vero and BHK-21 cell lines were inoculated with CHIKV at an MOI of 10^{-4}. Culture supernatants and infected cells were collected at 0, 10, 22, 34, 46, 70, and 106 hours. (***1***) Plaques were counted in plates previously seeded with 70.000 cells/mL incubated with the collected medium for five days. (***2***) RNA from infected cells was extracted, and the nsP1 CHIKV gene was amplified by RT-qPCR.

Table 1: Sample datasets were used to calculate the Chikungunya infectious titer by the Reed–Muench formula

**Dilution**		10^{-1}	10^{-2}	10^{-3}	10^{-4}	10^{-5}	10^{-6}	10^{-7}	10^{-8}	10^{-9}	10^{-10}	Mock	
**Scored cytopathic effect (CPE)**	**A**	CPE	CPE	CPE	CPE	CPE	NON CPE	NON CPE	NON CPE	NON CPE	NON CPE	NON CPE	NON CPE
	B	CPE	CPE	CPE	CPE	CPE	CPE	NON CPE	NON CPE	NON CPE	NON CPE	NON CPE	NON CPE
	C	CPE	CPE	CPE	CPE	CPE	NON CPE	NON CPE	NON CPE	NON CPE	NON CPE	NON CPE	NON CPE
	D	CPE	CPE	CPE	CPE	CPE	CPE	NON CPE	NON CPE	NON CPE	NON CPE	NON CPE	NON CPE
	E	CPE	CPE	CPE	CPE	CPE	NON CPE	NON CPE	NON CPE	NON CPE	NON CPE	NON CPE	NON CPE
	F	CPE	CPE	CPE	CPE	CPE	NON CPE	NON CPE	NON CPE	NON CPE	NON CPE	NON CPE	NON CPE
	G	CPE	CPE	CPE	CPE	CPE	CPE	NON CPE	NON CPE	NON CPE	NON CPE	NON CPE	NON CPE
	H	CPE	CPE	CPE	CPE	CPE	CPE	NON CPE	NON CPE	NON CPE	NON CPE	NON CPE	NON CPE

Table 2: Ct values were obtained from RNA isolated from Vero and BHK-21 cell lines infected with CHIKV at MOI of 10^{-4} at 0, 10, 22, 34, 46, 70, and 106 hours

	**Vero**				**BHK-21**			
Time	Ct_1	Ct_2	Ct_3	Ct_4	Ct_1	Ct_2	Ct_3	Ct_4
0	UD*	UD	UD	UD	UD	UD	UD	UD
10	36.448	36.363	36.326	36.528	32.209	32.581	32.317	32.992
22	33.832	33.431	33.990	33.359	30.261	30.494	30.232	30.297
34	28.225	29.559	28.268	28.431	25.631	25.691	25.929	25.450
46	24.711	24.081	24.734	24.297	22.011	22.651	22.647	22.732
70	22.347	22.980	22.253	22.834	20.935	20.448	20.284	20.690
106	21.811	21.800	21.945	21.830	20.864	20.617	20.597	20.270

* UD: undetermined

EXCLI Journal 2023;22:716-731 – ISSN 1611-2156
Received: June 08, 2023, accepted: July 20, 2023, published: July 27, 2023

Raw data concerning **Table 1:** Cytotoxic activity of *Marcetia taxifolia* flavonoids on BHK-21 cell line

Table 3: Absorbance values obtained by an MTT assay in BHK-21 cells infected with CHIKV and incubated with myricetin rhamnoside (MR), 5-hydroxy-3,6,7,8,3',4'-hexamethoxyflavona (HMF), 5,3'-dihydroxy-3,6,7,8,4'-pentamethoxyflavona (PMF) or myricetin 3-(6-rhamnosilgalactoside) (MRG) during 24, 48 and 72 hours

**MR**											
Concentration (µg/mL)											
	250	*125*	*62.5*	*31.3*	*15.6*	*7.81*	*3.9*	*1.95*	*0.97*	*0.48*	*0*
24 h	1.06	1.04	0.96	1.05	1.09	1.12	0.88	0.97	1.02	1.05	0.89
	1.01	1.13	1.22	0.86	0.98	0.78	0.81	0.93	0.96	0.94	0.83
	1.16	1.16	1.11	0.98	0.96	0.98	0.83	0.84	1.01	0.91	0.91
48 h	0.97	1.36	1.49	1.40	1.45	1.53	1.56	1.55	1.48	1.50	1.52
	0.87	1.41	1.31	1.29	1.36	1.50	1.38	1.44	1.47	1.27	1.47
	0.94	1.33	1.37	1.27	1.44	1.45	1.39	1.36	1.53	1.38	1.48
72 h	1.87	1.77	2.11	2.24	2.23	2.12	2.33	2.33	2.32	2.25	2.32
	1.74	1.66	1.87	1.88	2.18	1.97	1.93	2.12	2.21	2.13	2.03
	1.52	1.67	1.99	1.80	2.27	1.92	2.04	1.85	2.24	2.07	2.08
**HMF**											
Concentration (µg/mL)											
	250	*125*	*62.5*	*31.3*	*15.6*	*7.81*	*3.9*	*1.95*	*0.97*	*0.48*	*0*
24 h	0.11	0.11	0.77	0,64	0.50	0.41	0.55	0.67	0.81	0.72	0.73
	0.11	0.11	0.95	0,63	0.49	0.43	0.45	0.55	0.73	0.66	0.71
	0.11	0.11	0.66	0,61	0.55	0.47	0.52	0.69	0.62	0.94	0.83
48 h	0.11	0.11	0.28	0.50	0.66	0.68	0.80	0.92	1.09	1.19	1.22
	0.11	0.11	0.32	0.48	0.64	0.67	0.86	0.96	1.13	1.16	1.26
	0.11	0.13	0.21	0.38	0.58	0.81	1.04	1.23	1.29	1.45	1.54
72 h	0.07	0.07	0.38	0.61	0.85	0.87	1.34	1.57	1.69	1.88	1.88
	0.07	0.06	0.27	0.52	0.85	0.90	1.32	1.58	1.76	1.93	1.85
	0.10	0.06	0.44	0.63	0.88	0.97	1.53	1.89	1.84	1.98	1.89
**PMF**											
Concentration (µg/mL)											
	250	*125*	*62.5*	*31.3*	*15.6*	*7.81*	*3.9*	*1.95*	*0.97*	*0.48*	*0*
24 h	2.04	0.57	0.73	0.69	0.79	0.82	0.76	0.98	1.04	1.04	0.94
	0.37	0.48	0.60	0.67	0.63	0.89	0.88	0.95	1.06	0.96	0.90
	0.33	0.63	0.77	0.80	0.75	0.66	0.88	0.95	0.86	0.92	0.79
48 h	0.18	0.26	0.37	0.82	1.34	1.74	1.75	1.64	1.88	2.01	1.53
	0.18	0.28	0.39	0.95	1.40	1.72	1.75	1.72	1.79	1.92	1.71
	0.17	0.26	0.37	0.95	1.23	1.56	1.72	1.64	1.72	1.87	1.62
72 h	0.14	0.35	0.40	1.89	2.26	3.15	2.95	2.94	3.04	2.56	2.77
	0.12	0.24	0.40	1.85	2.37	2.71	2.61	2.51	1.98	2.20	2.58
	0.12	0.26	0.33	1.65	2.21	2.71	2.49	2.46	2.27	2.20	2.45

EXCLI Journal 2023;22:716-731 – ISSN 1611-2156
Received: June 08, 2023, accepted: July 20, 2023, published: July 27, 2023

**MRG**											
	Concentration (µg/mL)										
	250	*125*	*62.5*	*31.3*	*15.6*	*7.81*	*3.9*	*1.95*	*0.97*	*0.48*	*0*
24 h	0.20	0.91	1.12	1.01	0.96	0.93	0.86	0.98	0.93	0.95	0.88
	0.17	0.93	1.09	1.07	0.96	0.88	0.93	0.88	0.95	1.01	1.02
	0.16	0.83	1.10	0.98	0.98	0.99	1.15	1.07	1.12	1.06	0.98
48 h	0.21	1.14	1.62	1.87	1.68	1.70	1.58	1.50	1.58	1.68	1.54
	0.18	1.13	1.67	1.85	1.64	1.58	1.55	1.52	1.59	1.60	1.37
	0.19	1.13	1.52	1.74	1.52	1.60	1.50	1.52	1.67	1.66	1.63
72 h	0.15	1.49	2.67	2.66	2.47	2.23	2.31	2.28	2.30	2.05	2.43
	0.14	1.50	2.65	2.49	2.35	2.51	2.54	2.59	2.52	2.29	2.34
	0.14	1.51	2.27	2.76	2.29	2.49	1.56	0.75	0.95	1.72	2.56

EXCLI Journal 2023;22:716-731 – ISSN 1611-2156
Received: June 08, 2023, accepted: July 20, 2023, published: July 27, 2023

Raw data concerning **Figure 2:** BHK-21 cells infected with CHIKV were exposed to serial dilutions starting from 150 to 1.17 µg/mL for MR and 75 to 0.58 µg/mL for MRG for 72 hours. (***4***) Viral titer was obtained after five days of cell incubation with the supernatant assay medium. (***5***) nsP1 viral RNA copies from infected cells incubated with MR or MRG were amplified by RT-qPCR. Myricetin 3-(6-rhamnosilgalactoside) (MRG), myricetin rhamnoside (MR).

Table 4: Chikungunya infectious titers expressed as TCID50/mL according to Reed and Muench formula obtained after 72 h of the interaction of BHK-21 cell line with CHIKV at MOI of 10^{-4} and *M. taxifolia* compounds. Myricetin 3-(6-rhamnosilgalactoside) (MRG), myricetin rhamnoside (MR)

Treatment	*Rep* *	TCID50/mL
Initial titer	1	$10^{7.67}$
	2	$10^{8.33}$
MR 75µg/mL	1	$10^{5.33}$
	2	$10^{5.5}$
MR 37.5µg/mL	1	$10^{6.33}$
	2	$10^{6.17}$
MR 18.7µg/mL	1	$10^{6.33}$
	2	$10^{6.0}$
MR 9.3µg/mL	1	$10^{6.50}$
	2	$10^{6.17}$
MR 4.68µg/mL	1	$10^{7.33}$
	2	$10^{6.50}$
MR 2.43µg/mL	1	$10^{6.50}$
	2	$10^{6.67}$
MR 1.17µg/mL	1	$10^{6.67}$
	2	$10^{6.50}$
MR 0.58µg/mL	1	$10^{7.33}$
	2	$10^{6.50}$
MRG 75µg/mL	1	$10^{6.67}$
	2	$10^{7.33}$
MRG 37.5µg/mL	1	$10^{6.50}$
	2	$10^{6.50}$
MRG 18.7µg/mL	1	$10^{6.50}$
	2	$10^{6.67}$
MRG 9.3µg/mL	1	$10^{6.0}$
	2	$10^{6.0}$
MRG 4.68µg/mL	1	$10^{6.50}$
	2	$10^{7.17}$
MRG 2.43µg/mL	1	$10^{6.33}$
	2	$10^{6.50}$
MRG 1.17µg/mL	1	$10^{6.50}$
	2	$10^{7.0}$
MRG 0.58µg/mL	1	$10^{7.33}$
	2	$10^{6.67}$

Table 5: Ct values were obtained from RNA isolated from VERO and BHK-21 cell lines infected with CHIKV at MOI of 10^{-4} at 0, 10, 22, 34, 46, 70, and 106 hours. Myricetin 3-(6-rhamnosilgalactoside) (MRG), myricetin rhamnoside (MR)

	**MR**			**MRG**	
**Concentration (µg/mL)**	Ct_1	Ct_2	**Concentration (µg/mL)**	Ct_1	Ct_2
150	36.298	36.186	75	30.557	30.505
75	26.052	25.870	37.5	24.399	24.833
37.5	25.524	25.586	18.75	23.971	23.706
18.75	24.660	24.723	9.37	23.117	23.071
9,37	24.080	23.946	4.68	22.396	22.850
4.68	23.745	23.780	2.34	22.006	22.063
2.34	21.991	22.055	1.17	21.389	21.454
1.17	21.405	21.683	0.58	20.517	20.378
0	UD*	UD	0	UD	UD

* UD: undetermined

Raw data concerning **Figure 3** of molecular docking assays of Myricetin 3-(6-rhamnosilgalactoside) (MRG) and myricetin rhamnoside (MR) with nsP3 are available in the ASUS WebStorage cloud storage service at the link: https://www.asuswebstorage.com/navigate/a/#/s/09DD67A0FF82447985A0CEA05C88675C4

Raw data concerning **Figure 4** of Molecular dynamics of Myricetin 3-(6-rhamnosilgalactoside) (MRG) and myricetin rhamnoside (MR)-nsP3 complex are available in the ASUS WebStorage cloud storage service at the link: https://www.asuswebstorage.com/navigate/a/#/s/AAD424377158484993A1D09CF38A2A6F4
