# Supplementary material for: Antiviral activity of myricetin glycosylated compounds isolated from Marcetia taxifolia against chikungunya virus
Source: EXCLI J. 2023 Jul 27;22:716–31. doi: 10.17179/excli2023-6242 (PMC10471840; doi:10.17179/excli2023-6242)
Supplement: Supplementary information [file EXCLI-22-716-s-002.pdf]

EXCLI Journal 2023;22:716-731 – ISSN 1611-2156
Received: June 08, 2023, accepted: July 20, 2023, published: July 27, 2023

Supplementary information to:

Original article:

ANTIVIRAL ACTIVITY OF MYRICETIN GLYCOSYLATED COMPOUNDS ISOLATED FROM *MARCETIA TAXIFOLIA* AGAINST CHIKUNGUNYA VIRUS

Ana Luisa Muñoz[1], Andrés Felipe Cuéllar[1], Gabriela Arévalo[1], Brian David Santamaría[1], Anny K. Rodríguez[1], Cristian Buendia-Atencio[1], Andrés Reyes Chaparro[2], Aldo Yair Tenorio Barajas[3], Nidya Alexandra Segura[4], Felio Bello[5], Alírica I. Suárez[6], Héctor R. Rangel[7], Monica Losada-Barragán[1*]

[1] Faculty of Science, Universidad Antonio Nariño (UAN), Bogotá 110231, Colombia
[2] Escuela Nacional de Ciencias Biológicas (ENCB), Departamento de Morfología, del Instituto Politécnico Nacional (IPN), Mexico
[3] Facultad de Ciencias Físicomatemáticas, Benemérita Universidad Autónoma de Puebla C.U. Puebla, Puebla, Mexico
[4] Faculty of Science, Universidad Pedagógica y Tecnológica de Colombia, Tunja 150003, Colombia
[5] Faculty of Agricultural and Livestock Sciences, Program of Veterinary Medicine, Universidad de La Salle, Bogotá 110141, Colombia
[6] Natural Products Laboratory, Faculty of Pharmacy, Universidad Central de Venezuela, Caracas, Venezuela
[7] Molecular Virology Laboratory, Instituto Venezolano de Investigaciones Científicas, Caracas, Venezuela

* **Corresponding author:** Dra. Monica Losada-Barragán, Universidad Antonio Nariño-Sede Circunvalar. Cra. 3 este # 47A - 15, Bogotá, Colombia.
E-mail: monica.losada@uan.edu.co

https://dx.doi.org/10.17179/excli2023-6242

This is an Open Access article distributed under the terms of the Creative Commons Attribution License (http://creativecommons.org/licenses/by/4.0/).

EXCLI Journal 2023;22:716-731 – ISSN 1611-2156
Received: June 08, 2023, accepted: July 20, 2023, published: July 27, 2023

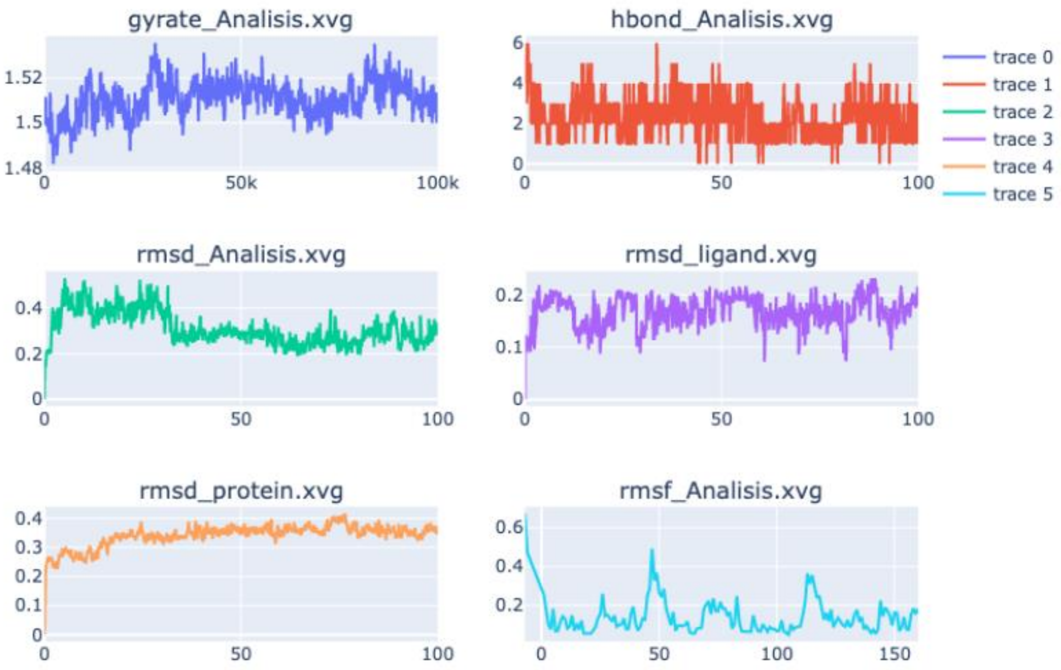

Supplementary Figure 1: Molecular dynamics of MR-nsP3 complex

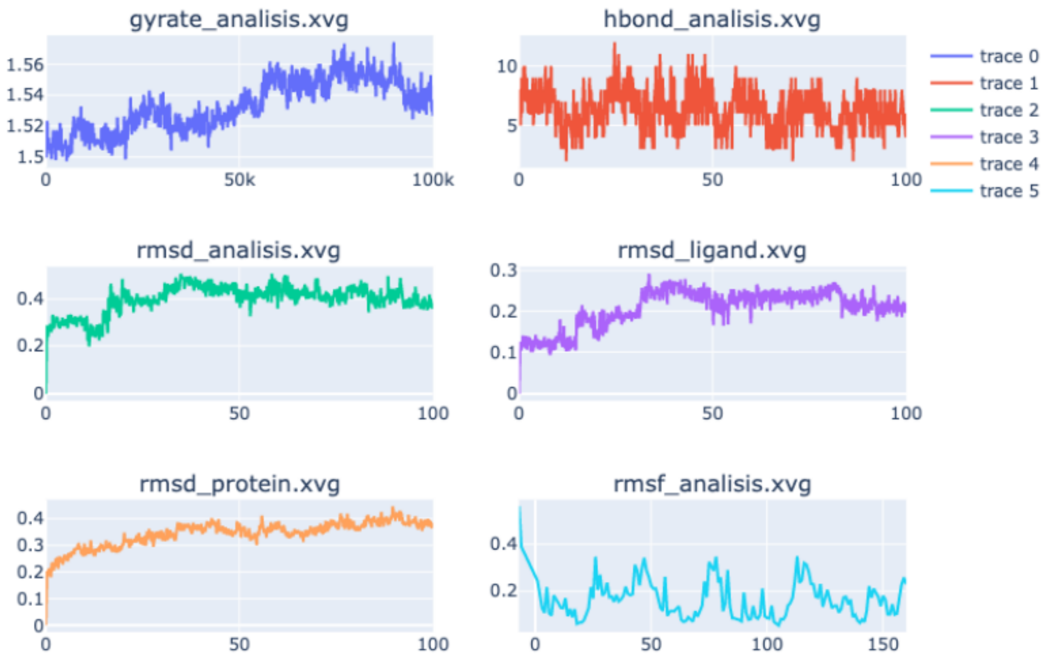

Supplementary Figure 2: Molecular dynamics of MRG-nsP3 complex
